# Utility and health-related quality of life measures in adult Colombian patients with solid tumours

**DOI:** 10.3332/ecancer.2021.1240

**Published:** 2021-06-03

**Authors:** Rafael Chaves-Cardona, Martín Romero Prada, Maria Victoria Ocampo, Duván Gallo, Lina María Gómez, Natalia Clavijo

**Affiliations:** 1 Jorge Tadeo Lozano University, Calle 127a N 70h-42, Bogotá, Colombia; 2 Proyéctame Group, Transversal 60#124-20 Oficina 210, Colombia

**Keywords:** quality of life, patient reported outcomes, cancer

## Abstract

According to a 2020 report, the World Health Organization explained how, in 20 years, the prevalence of cancer cases will increase by 60% worldwide. In lower-middle-income countries, this figure will be 74.07%. Therefore, the authors propose a series of recommendations, such as how to address both traditional health indicators and the psychosocial environment, to improve the health system. The objective of this study is to demonstrate the impact of cancer on the quality of life (QoL) and health status of oncology patients in Colombia. An observational cross-sectional study using patient reported outcomes tools, such as European Organization for Research and Treatment of Cancer (EORTC) Quality of Life of Cancer Patients (QLQ-C30) and EuroQoL-5 dimensions questionnaire-3 levels (EQ5D-3L), was carried out. The information of 356 people was compiled. They were contacted by patient associations. The results were analysed using descriptive and inferential statistics, using ordinary least squares methodology. For the EORTC QLQ-C30, overall health status was 66.05 (95% confidence interval: 63.78–68.32), on the functional scales, emotional and social function were the two scales with the lowest ratings (71.57 and 71.77), without any representative differences. For the EQ5D-3L, the average utility was 0.70 (Standard deviation: 0.20); 50% of people had a utility between 0.63 and 0.82. The analysed population was most affected in the following areas: financial difficulties, insomnia, anxiety, depression and emotional functioning, establishing the need for future interventions and the creation of public policies that generate a better QoL for patients.

## Introduction

According to a 2018 report by the Cuenta de Alto Costo (the Colombian fund for high-cost diseases), the reported prevalence of cancer in the Columbian population was 275,348, of which 37,630 were new cases. This was an overall increase of 15.82% compared to 2017. Additionally, 95.1% of the prevalent cases were of an invasive nature and 4.9% were *in situ* cancers, a similar behaviour to the incident cases reported [1]. The prevalence of invasive cancer has increased from 393.3 in 2016; 446.9 per 100,000 inhabitants in 2017; to 531 per 100,000 inhabitants in 2018 [1, 2]. In a 2020 report, the World Health Organization (WHO) explained how, in the next two decades, the prevalence of cancer will increase by 60% worldwide, and in lower-middle-income countries, such as Colombia, an increase of 74.07% could occur [3].

Beyond the WHO preventative measures for the reduction of incident cases, this body sets out as its main recommendation, improvement in the provision of services for oncology patients [3] including creating policies appropriate to each country [4], which should address not only traditional health indicators but also the psychosocial environment [5]. Whatever the cause for the increase in this condition, it has made cancer a priority for Colombia’s public health policies. In this regard, the government has set out actions for the comprehensive care of cancer patients, framed within the 10-year plan for the control of cancer 2012 to 2021 [6], and in law 1384 of 2010 [7], which sets out that it is essential to improve the quality of life (QoL) of patients. Despite having the objective of providing adequate information on cancer for decision-making, the National Cancer Observatory (ONC Colombia) does not address the areas of physical, mental and social well-being in their entirety. They do little to study the results in depth. Consequently, their actions related to the improvement of the QoL for cancer patients are limited. They also fail to mention the results to patients as a main source of information [8].

Conceptually, health-related quality of life (HRQOL) facilitates an understanding of the patient's well-being from their own point of view. This enables evaluation of any changes resulting from medical interventions or health systems [9]. Thereby, demonstrating its utility for describing the impact of the disease on patients’ lives [10]. In the same way, the concept of patient reported outcomes (PRO) is the information provided by the patient, explaining the symptoms associated with their health status and how they affect his or her HRQOL [11]. In other words, HRQOL is a concept that includes multiple dimensions representing the patient’s overall perception of the effects of the disease and treatment of different aspects of their physical, psychological and social life. Such information provided by the patient will be the basis for the proper design of the care pathway, especially for patients with advanced cancer [12].

In this sense, the systematic implementation of questionnaires that evaluate the PRO of cancer patients could contribute to the comprehensive care proposed by the national government. This is especially the case in the care of patients with advanced cancer, such as that developed in palliative care models, where an organised intervention for predominant symptoms improves HRQOL. Therefore, as an initial step towards the development of these strategies, this study proposes to seek to demonstrate the utility of this type of questionnaire for patient care in Colombia [10, 11].

There are some scientific publications evaluating QoL of patients with cancer in Colombia. Castillo-Ávila *et al* [13] carried out one such study on a group of people with cervical cancer. They were subject to the application of PRO tools, such as the European Organization for Research and Treatment of Cancer (EORTC) Quality of Life of Cancer Patients (QLQ-C30) and the EORTC QOL-Quality of Life of Cervical Cancer. This showed the most affected aspects of QoL related to health were: social activities (51.9%), functional and physical status, effects of treatment on family life (48.1%) and financial difficulties as a result of the disease (47.2%) [13]. In Antioquia, Salas and Grisales [14] using the WHOQOL BREF (World Health Organization Quality of Life, abbreviated version) demonstrated that socioeconomic factors, level of education, family support and cultural and religious beliefs affect the QoL of women being treated for breast cancer. The study carried out by Gaviria *et al* identified significant levels of anxiety and depression in oncology patients through the use of PRO tools [15]. Mejía-Rojas *et al* [16] identified how the effects of treatment for patients with breast cancer were the main aspects that led to a deterioration in the QoL, using tools such as QLQ-C30 and Quality of Life Breast Cancer.

This study forms part of the PROCáN strategy, a support for the implementation of public health activities, focused on measuring the health status of cancer patients. It is used to generate valid information on critical issues requiring intervention, and to then plan multidimensional interventions that improve the QoL of these patients. As a starting point for the design of the strategy, it was necessary to have an initial measurement of the perceived outcomes for cancer patients and the changes in respect of their conditions. This study aims to demonstrate the impact of cancer on QoL and health status in Colombia, the utility of this type of questionnaire for the care of cancer patients, and especially for those in the advanced stages of the disease. The questionnaires are expected to provide useful information to develop proposals and actions for public policy for the care of cancer patients in the country.

## Methodology

A joint strategy, called PROCáN, was developed by a group of social and health science researchers, with the support of cancer patient associations, in different cities of the country, and was coordinated by the Fundación Simmon (a Columbian cancer foundation). This joint strategy developed a cross-sectional study to evaluate the perceived QLQ-C30 in Colombia. It was approved by the ethics committee.

The Spanish versions of the European Organization for Research and Treatment of Cancer (EORTC) Quality of Life of Cancer Patients (QLQ-C30) [17] and the EuroQoL-5 dimensions questionnaire-3 levels (EQ5D-3L) [18] questionnaires were used to assess QoL and health status. From a review of the literature, it was identified that the EORTC QLQ-C30 is one of the most widely used questionnaires, because it can be used for patients with any type of cancer; in addition, there is a validated version for use in Colombia [17]. This is a multidimensional questionnaire with 30 questions, which evaluate five aspects of QoL (physical, role, cognitive, emotional and social), three symptom scales (fatigue, pain, nausea and vomiting), a global health status scale (QoL) and single items assessing additional, commonly reported symptoms (dyspnoea, loss of appetite, insomnia, constipation and diarrhoea) [19]. The five aspects are assessed on a scale of 1–100, where 1 reflects a lower perception of health and 100 a higher perception. On the symptom scale, the assessment is also from 0 to 100 but inversely, 1 indicates that the symptom is not present and 100 represents a poor presentation of the symptom. Assessment of the EORTC QLQ-C30 was carried out using the recommendations from the EORTC data centre [19].

The second tool used was the EQ5D-3L questionnaire [18] in Spanish validated for Colombia, which was developed by the EuroQoL group [20]. It is used to identify health status, measure utility and evaluate HRQOL. It consists of two components: the first evaluates five dimensions: mobility, self-care, usual activities, pain/discomfort and anxiety/depression. Each of the dimensions is evaluated at three levels of severity: 1 = no problems; 2 = some problems and 3 = extreme problems. This scale gives a five digit number as a result, which describes the health status of the patient. It is possible to obtain up to 243 possible health statuses, with 11,111 being the best possible health status with a utility of 1, and 33,333 the worst possible status with a utility of −0.101 [21]. The second component is a visual analogue scale, in which the patient rates his or her health status, with 1 being the worst state imaginable and 100 the best. To assess health status using the EQ5D-3L, the recommended scoring for Colombia was used, obtained by Zarate *et al* [21].

The use of physical versions of the two questionnaires for this study in quantities greater than the estimated sample size, was authorised by the licence owners. The existence of the studies and their corresponding validation for Colombia were previously verified [22].

The sample size was estimated with a confidence level of 99%, the standard deviation taken from previous studies was 20 [23], and the data accuracy obtained from ±3 of the HRQOL measurements. An estimate was obtained using the calculation of a finite population of an estimated 198,798 [24] cancer patients in Colombia in 2016, with an expected loss of 15%, which gave 346 patients. The sample was gathered by patient associations who brought together patients in different cities around the country (Bogotá, Cali, Santa Marta, Valledupar and Villavicencio). This was coordinated by the Fundación Simmon. Only adult patients with a solid-type cancer and a confirmed diagnosis of cancer were included. Acceptance for inclusion in this study was validated through signed informed consent. The target population for this study participated freely and voluntarily, without any coercion over their responses; they were free not to answer any questions if they did not wish to do so.

The survey, which included characteristics of the patient and their disease, and the established questionnaires, was carried out by health personnel, who had previously received training on the objective of the study and the application of the tools. It was carried out during June and July 2018. The information was collected on paper. Then, it was transcribed into a single database. This process was validated by two of the researchers to avoid the risk of losing information and to ensure the quality of the data obtained.

The data on the characteristics of the population and their disease were analysed with descriptive statistics using the mean and standard deviation. Inferential statistics were also used for characteristics. CIs of 95% were taken and the scores obtained were compared with the QoL tools using ordinary least squares regression methodology; values of p < 0.05 were taken to be statistically significant. The statistical software package Stata Version 14 was used for the study [25].

## Results

A total of 355 people were surveyed. The data collection process was coordinated by the Fundación Simmon. The respondents had an average age of 56.12 years (Standard deviation (SD): ±14.3 years) and an interquartile range of 47–67 years. 69.01% of the patients were female, with 245 women taking part; 2.3 women were surveyed for every man participating (Table 1).

The most frequent types of cancer were breast, other and colon; nine individuals reported having more than one type of cancer, similar to that reported in the literature. Most patients were being treated with chemotherapy and radiotherapy. It was highlighted that 5.67% of the respondents did not know their status at the time of the survey (Table 2). To show the time that had elapsed from diagnosis of the disease to the survey date, the results are presented based on the information provided by the respondents. 82.53% of the population had a diagnosis date, so it was possible to identify how long the patient had had the disease, excluding 24 patients who did not remember their diagnosis date, and 60 patients who failed to record it fully (Table 2).

The results relating to the global health scale using EORTC QLQ-C30, from 0 to 100, with 100 being the best perception of health, measured overall health status as 66.05 (95% confidence interval (CI): 63.78–68.32). When functional aspects were evaluated, emotional and social functions had the lowest scores (71.57 and 71.77), without any representative differences between the functional scales. Within the symptom analysis, which is rated inversely, that is to say, the higher the score, the worse the perception of health, financial difficulties and insomnia were those that attracted the worst scores (58.2 and 36.76) (Table 3).

The analysis of the health status of those surveyed according to the responses of the EQ5D-3L tool took into account 338 respondents, as they fully responded to this part of the survey. It was found that the average utility was 0.70 (SD: 0.20); 50% of people had utilities between 0.63 and 0.82.

Depending on the different demographics and clinical characteristics, where QoL was concerned, there was a relationship between the average scores found by the EQ5D-3L and EORTC QLQ-C30 tools. Regarding analysis by gender, it was shown that with both tools, women scored worse in both the general components and the specific scales; this difference being statistically significant only for the utility of the EQ5D-3L. Additionally, the worst average score was among cancer patients with a pre-existing comorbidity, in those whose cancer had progressed, and those who did not know what their current status was, compared to those who had improved, this was a statistically significant difference (*p* < 0.05). No significant differences were identified by age, type of treatment in the last 6 months or by duration of disease (Table 4).

## Discussion

The results of this study demonstrate the utility of making a parametric assessment of cancer symptoms by showing the differences between patients. Additionally, the health situation of patients with more advanced stages of the disease was more compromised by the severity of the symptoms.

Studies previously conducted in Colombia, such as that of Bermúdez *et al* [22], where QoL was evaluated using the QLQ-C30 questionnaire in a cancer population which attended a foundation in the city of Bucaramanga, came up with an average score of 60 ± 9.3 (95% CI: 57.01–62.99). This was in accordance with the data obtained in this study, which identified an overall score of 66.05 (95% CI: 63.78–68.32).

Finck *et al* [23], using the EORTC QLQ-C30 on 1,500 Colombians in the general population, found an average score for overall health status of 77.1 (SD: 18.5). When this result is compared to the QoL measurement obtained in cancer patients included in this study, the decrease due to the disease is more than 10 points (an average of 66.05,95% CI: 63.8–68.3). The functional and symptom scales showed that the population with cancer was more affected than the general population. The general population had functional scale scores above 87, while in patients with cancer it was cognitive function that scored the highest with 76.8 (73.93–79.66). The symptom with the greatest impact on the general population was fatigue, with a score of 14.0 (SD: 18.4), while in the cancer population it was dyspnoea (average 13.68,95% CI: 10.89–16.46). In addition, participants in this analysis experienced more financial difficulties, reaching an average of 52.8 (95% CI: 48.68–56.92), while in the general population the average score is 7.2. By seeing these differences, the sensitivity of the questionnaire can be demonstrated by identifying the extent of complications that cancer patients experience compared to the general population, which is the objective of this research.

Regarding international studies, Hinz *et al* [26] conducted a QoL study with the EORTC QLQ-C30 tool among 2,059 German patients with different types of cancer. They found an average reduction in the functional scale of 11.8 points compared with the general German population (the average for the cancer population was 74.8, SD: 19.3), with a difference of 9.2 points for the symptom scale (the average for the cancer population was 20.8, SD: 16.6). When comparing it with the results of this study, there are similarities in the average score on the functional scale, being 73.81 (95% CI: 71.56–76.04) and for the symptom scale, being 29.02 (95% CI: 26.88–31.14). For the functional scales, it was shown for both the German population and the patients analysed in this study, that the emotional aspect was affected the most, with values of 69.8 (SD: 24.9) and 71.57 (95% CI: 68.74–74.40), respectively. The most important difference between the two studies was in the symptom scale, with the highest score being for fatigue in the German population (35.6, SD: 25.6), while in this study financial difficulty scored the highest (52.8 – 48.68 – 56.92).

In Latin America, Torrecilla *et al* [27] identify the needs of patients in clinical practice, from a perspective that includes psycho-oncology, optimising health services by correctly addressing the needs of 80 women diagnosed with cancer, including the measurement of QoL using the EORTC QLQ-30; financial difficulties were identified as one of the most affected items, as in this analysis. Similarly, the study by Salas and Grisales [14] in Antioquia, which evaluated QoL of patients with breast cancer using the WHOQOL-100 scale, found that women with the disease had a lower score in the physical, psychological and social domains, as well as a lower overall score. This demonstrates that the approach to oncology patients goes beyond the provision of health services and highlights the need to implement treatment strategies focused on the needs of the patients, addressing emotional aspects and the financial burden.

For this study, using both the EORTC QLQ-C30 and the EQ5D-3L scales, the anxiety, depression and emotional function aspects were those most affected. The Colombian health system does not provide an adequate response to these issues within its care routes. Among the surveyed patients, financial difficulties produced the worst assessment in terms of outcome. This highlights the need for specific new studies to determine, in more detail, the perceived financial toxicity, as this can vary in context from pocket money to the perception of social financial disaster, in addition to the corresponding effects of financial distress on the patient’s daily life, among other things.

Cancer, defined in Colombia as a catastrophic disease due to the technical complexity and high cost of its treatment, and the low cost-effectiveness of the interventions [28], generates a worse perception in patients' QoL, a situation that is reflected in this and in other published studies [14, 22]. Furthermore, a differing impact on the QoL of people with comorbidities and with progressive disease is shown, demonstrating that there are people with specific needs who must be considered when implementing additional measures to improve their health condition.

Current care models have focused on the definition of curative interventions, diagnostic methods and medication. This study demonstrates the importance of incorporating follow-up and interventions for symptoms that have not normally been recognised as indicators in patients with cancer, such as insomnia or financial toxicity. The aim is to promote the use of QoL measures using the patient as a primary source of information; to identify the most affected dimensions in patients with interesting pathology; to develop damage limitation processes; and to develop follow-up actions within the Colombian social security system that help evaluate the proposed strategies.

Regarding the limitations of this study, it should be taken into account that the information obtained is self-reported by patients who suffer from cancer and there is no detailed clinical description of the stages of the disease for each person. On the other hand, the objective of this analysis was not to find differences by specific phases or types of treatment. Only a descriptive approach was taken, whereby, information can be made known in the near future. Additionally, in the development of this study, it was impossible to use a questionnaire designed specifically for the Colombian population. This made it possible that dimensions that would have been of vital importance for Colombian patients, in accordance with their socio-cultural context, were not taken into account or evaluated. However, the EORTC QLQ-C30 questionnaire was used in the study, which was partly validated by Finck *et al* [23], who assessed the scale on the general population, aged over 18 years, in the major cities of Colombia.

It should be noted that within this study, it is possible that there is no representative sample by socioeconomic status, since cancer patients above level 5 (very high socioeconomic level) do not seek care in organisations such as foundations. However, considering that this population makes up less than 10%, the data presented in this analysis enables us to focus on the creation of public policy interventions which protect the majority of citizens.

The application of PRO tools succeeds in identifying areas for intervention, enabling their adjustment to the needs of the patient and developing their respective follow-up. Kaasa *et al* [29] explain the use of results reported by patients to improve symptom control, achieve better physical and mental health and make better use of health services, since there is no agreement that standardises priorities for the management of the palliative care patient. It is also pertinent to continue conducting future studies that address the QoL and other palliative needs of this population. In this study, it was demonstrated how patients who had stated that the cancer had progressed, had a lower QoL when compared to those who said that the cancer had improved, was under control or did not know, by recording lower average measures for EQ5D-3L, the overall health status and functional scale of the EORTC; and higher average measurements for the symptoms scale of the same PRO tool. This demonstrates the importance of intervention for these patients. One of the intervention strategies put forward by WHO [14] to improve the patient's QoL, alleviating physical, psychological, social or spiritual suffering [13] is to include the palliative needs of oncology patients [12]. This study demonstrates the benefit of its application to cancer patients at different stages of the disease, and demonstrates how its routine use, as part of clinical activity, would enable better targeting of actions, in groups where the presence of symptoms is even more sensitive, as could be the case in patients with advanced disease [16, 30].

In addition, it shows how cancer negatively impacts patients' QoL when viewed from the overall QoL perspective; such as the different dimensions that can be assessed when using PRO tools, as well as providing important information on care, it also demonstrated their sensitivity at different stages of the disease.

## Conclusions

Patients diagnosed with cancer experience deterioration in their QoL, presenting with effects in areas such as financial difficulties, insomnia, anxiety, depression and emotional function, in addition to the symptoms related to the disease and its treatment. The use of specific assessment tools, such as the EORTC QLC30, enables a parametric identification of multidisciplinary care and standardisation of habitual use, which could be the subject for public policy.

## List of abbreviations

HRQOL, Health-related quality of life; SD, Standard deviation; EORTC, European Organization for Research and Treatment of Cancer; EQ5D-3L, EuroQoL-5 dimensions questionnaire-3 levels; CI, Confidence interval; WHO, World Health Organization; PRO, Patient reported outcome; QLQ-C30, Quality of life of cancer patients; WHOQOL BREF, World Health Organization quality of life, abbreviated version; WHOQOL-100, World Health Organization quality of life.

## Ethics approval and consent to participate

This study was approved by the Riesgo de Fractura (fracture risk) ethics committee, study number 30983. For all participants, written consent was obtained in accordance with the study protocol.

## Conflicts of interest

The researchers participating in this research project have not declared any conflicts of interest.

## Funding

The PROCáN project received funding for its implementation through a grant from MSD laboratories. However, this did not influence the design, analysis or results of the study.

## Figures and Tables

**Table 1.** Socio-demographic characteristics of the surveyed population.

Characteristic	*n*	%
**Age**		
18–44 years	72	20.28
45–59 years	128	36.06
60–69 years	90	25.35
70 and older	65	18.31
**Gender**		
Female	245	69.01
Male	108	30.42
Unknown	2	0.56
**Marital status**		
Married	134	37.75
Divorced	24	6.76
Other	15	4.23
Single	87	24.51
Civil partner	70	19.72
Widow(er)	25	7.04
**Level of education**		
Postgraduate	7	1.97
Professional	32	9.01
Technical	52	14.65
Baccalaureate	123	34.65
Primary school	120	33.80
None	20	5.63
No information	1	0.28
**Household**		
Partner/children	262	73.80
Alone	25	7.04
Friends	1	0.28
Other/family	65	18.31
No information	2	0.56
**Employment status**		
Seeking work	8	2.25
Student	7	1.97
Unemployed	138	38.87
Pensioner	37	10.42
Works at home	93	26.20
Worker	69	19.44
No information	3	0.85

**Table 2.** Clinical characteristics of the respondents.

Characteristic	*N*	%
**Type of cancer**		
Colorectal	35	9.62
Cervical	31	8.52
Gastric or stomach	21	5.77
Skin	13	3.57
Lung	19	5.22
Prostate	31	8.52
Other	67	18.41
Breast	147	40.38
**Presence of comorbidities**		
Yes	145	40.84
No	210	59.15
**Current treatment**		
Chemotherapy	241	66.39
Radiotherapy	37	10.19
Surgery	3	0.83
Other	30	8.26
None	48	13.22
**Treatment within the last 6 months**		
Chemotherapy	192	56.30
Radiotherapy	72	21.11
Surgery	40	11.73
Other	15	4.40
None	22	6.45
**Time since diagnosis of disease**		
Less than 1 year	13	4.44
1 year	150	51.19
2 years	51	17.41
3 years	26	8.87
4 years	13	4.44
5 years or more	40	13.65
**Stage of the disease**		
Controlled	177	50.14
Has improved	96	27.20
Has progressed	60	17.00
Unknown	20	5.67

**Table 3.** QoL assessment using EORTC QLQ-C30. Bold values are statistically significant to 95% interval confidence.

Dimension	EORTC QLQ-C30	CI
**Overall health status**	**66.05**	**(63.78–68.32)**
**Functional scale**	**73.81**	**(71.56–76.04)**
Physical	74.72	(72.15–77.29)
Role	75.45	(72.29–78.59)
Emotional	71.57	(68.74–74.40)
Cognitive	76.80	(73.93–79.66)
Social	71.77	(68.46–75.07)
**Symptoms scale**	**29.02**	**(26.88–31.14)**
Fatigue	35.81	(32.96–38.64)
Nausea and vomiting	16.48	(13.90–19.04)
Pain	31.49	(28.26–34.71)
Dyspnoea	13.68	(10.89–16.46)
Insomnia	36.76	(33.16–40.36)
Loss of appetite	29.46	(25.86–33.05)
Constipation	22.10	(18.95–25.23)
Diarrhoea	17.77	(14.91–20.61)
Financial difficulties	52.80	(48.68–56.92)

**Table 4.** EORTC QLQ-C30-EQ5D-3L: Descriptive and inferential analysis.

			EORTC QLQ-C30					
	EQ5D-3L		Overall health status		Functional scale		Symptoms scale	
Characteristic	Avg (SD)	*p*-value	Avg (SD)	*p*-value	Avg (SD)	*p*-value	Avg (SD)	*p*-value
**Gender**								
Female	0.68 (0.2)	-	64.45 (22.39)	-	72.02 (22.03)	-	30.51 (20.8)	-
Male	0.76 (0.2)	0.24	69.7 (19.37)	0.414	78.03 (19.27)	0.569	25.43 (19.04)	0.934
**Age**								
18–44 years	0.71 (0.18)	-	65.96 (18.94)	-	73.94 (18.47)	-	27.8 (18.86)	-
45–59 years	0.7 (0.19)	0.447	67.72 (21.67)	0.453	74.36 (21.86)	0.493	28.55 (19.85)	0.252
60–69 years	0.7 (0.22)	0.543	65.56 (22.49)	0.982	74.18 (22.16)	0.614	29.77 (20.92)	0.167
70 and older	0.7 (0.22)	0.497	63.59 (23.64)	0.933	72.03 (23.09)	0.698	30.24 (22.61)	0.329
**Treatment within the last 6 months**								
Chemotherapy	0.70 (0.2)	0.961	64.69 (20.4)	0.431	74.07 (19.77)	0.493	31.19 (20.43)	0.661
Radiotherapy	0.69 (0.2)	0.512	65.49 (19.79)	0.663	73.50 (23.05)	0.620	30.44 (23.17)	0.262
Surgery	0.68 (0.19)	0.344	58.97 (20.62)	0.193	68.05 (17.82)	0.226	32.87 (21.13)	0.557
Other	0.71 (0.22)	0.882	64.49 (25.77)	0.787	72.47 (26.74)	0.575	28.92 (23.54)	0.207
None	0.71 (0.24)	0.314	66.67 (28.05)	0.143	67.61 (26.81)	0.866	27.12 (23.33)	0.017^a^
**Disease duration**								
Less than 1 year	0.68 (0.27)	-	64.74 (21.29)	-	**65.46 (32.7)**	-	35.31 (28.94)	-
1 year	0.70 (0.18)	0.764	67.39 (20.39)	0.972	**74.91 (19.86)**	0.371	28.38 (19.67)	0.413
2 years	0.72 (0.21)	0.439	66.17 (22.86)	0.997	**74.96 (19.89)**	0.334	29.53 (19.2)	0.602
3 years	0.67 (0.22)	0.764	66.03 (23.56)	0.658	**73.54 (22.46)**	0.506	29.43 (23)	0.468
4 years	0.77 (0.19)	0.217	67.31 (25.79)	0.995	**79.66 (17.45)**	0.055	24.9 (19.09)	0.198
5 years or more	0.71 (0.16)	0.200	62.92 (24.75)	0.835	**72.89 (22.44)**	0.153	27.99 (17.64)	0.144
**Presence of comorbidities**								
Yes	0.67 (0.2)	0.002^a^	65.1 (22.41)	0.148	70.65 (22.60)	0.008^a^	31.81 (20.13)	0.006^a^
No	0.73 (0.2)	-	66.71 (21.24)	-	75.98 (20.40)	-	27.09 (20.39)	-
**Stage of the disease**								
Has improved	0.75 (0.16)	-	71.79 (19.51)	-	78.88 (19.13)	-	24.47 (17.83)	-
Controlled	0.75 (0.18)	0.628	68.8 (19.61)	0.280	77.96 (17.98)	0.878	24.7 (17.2)	0.518
Has progressed	0.52 (0.22)	0.000^a^	52.26 (24.31)	0.000^a^	57.15 (23.73)	0.000^a^	45.15 (22.55)	0.000^a^
Unknown	0.63 (0.19)	0.032^a^	55 (22.2)	0.001^a^	64.15 (24.91)	0.177	40.36 (23.52)	0.007^a^
**Type of cancer**								
**Colorectal**	0.75 (0.19)	-	**69.89 (21.48)**	-	79.21 (19.54)		25.29 (19.32)	-
**Cervical**	0.63 (0.29)	0.258	61.41 (24.69)	0.496	68.77 (27.91)	0.327	35.47 (26.89)	0.251
**Gastric or stomach**	0.72 (0.27)	0.942	72.91 (20.06)	0.936	79.36 (18.86)	0.967	29.02 (18.65)	0.621
**Skin**	0.82 (0.15)	0.59	78.84 (13.01)	0.140	83.85 (14.69)	0.496	16.28 (16.71)	0.048^a^
**Lung**	0.68 (0.24)	0.970	**52.78 (30.52)**	0.057	64.89 (29.60)	0.317	39.77 (28.56)	0.508
**Prostate**	0.76 (0.22)	0.661	68.82 (15.06)	0.638	77.60 (20.77)	0.939	22.69 (12.61)	0.356
**Other**	0.67 (0.22)	0.283	63.25 (23.88)	0.413	66.89 (24.11)	0.142	34.09 (23.36)	0.278
**Breast**	0.70 (0.14)	0.614	66.32 (20.27)	0.938	75.39 (17.87)	0.493	27.40 (17.21)	0.730

The *p*-value was obtained using the ordinary least squares regression methodology. Avg, Average; SD, Standard deviation

a Statistically significant differences, value of *p* < 0.05
